# Trend of HIV Prevalence in Antenatal Women in a Tertiary Care Hospital in the Western Region of India

**DOI:** 10.7759/cureus.57125

**Published:** 2024-03-28

**Authors:** Zalak V Karena, Disha Padaliya, Dhruvin S Viradia

**Affiliations:** 1 Obstetrics and Gynecology, GMERS (Gujarat Medical Education & Research Society) Medical College, Morbi, IND; 2 ART (Anti-Retroviral Treatment) Centre, GMERS (Gujarat Medical Education & Research Society) Medical College, Morbi, IND

**Keywords:** hiv viral load, anti-retroviral therapy (art), trend, prevalence, antenatal, hiv

## Abstract

Background

The severity of AIDS and the social and personal implications of HIV makes the epidemiological study of HIV more difficult. Surveillance studies remain the mainstay of understanding the trends of HIV infection. In this study, we aimed to determine the trend in the prevalence of HIV in the antenatal women attending our hospital situated in the western region of India over the period of the last nine years and the ART adherence and the viral loads of the cluster of positive patients identified from this antenatal HIV testing.

Methodology

A retrospective study was conducted by collecting data for nine years from January 2015 to December 2023 from the PPTCT (Prevention of Parent to Child Transmission) Centre and ART (Anti-Retroviral Treatment) Centre of the hospital. All pregnant women attending antenatal clinics and being admitted to the labor room are counseled for HIV testing as per the National AIDS Control Organisation (NACO) guidelines of India. The data of the total antenatal women counseled for HIV testing and who tested HIV positive were collected. The HIV prevalence rate was derived and the trend of HIV prevalence in antenatal women attending the hospital was determined over the study period. The data on ART adherence and the viral load of these HIV-positive women detected antenatally and their seropositive spouse and children were collected and analyzed.

Results

A total of 22,584 antenatal women were counseled for HIV testing during the study period. No women opted out and there was 100% testing of these 22,584 antenatal women for HIV. Fifty antenatal women tested positive for HIV, resulting in an overall HIV prevalence of 0.22% (50/22,584) during the study period. There was a declining trend of HIV prevalence among antenatal women from 2020 to 2023 (from 0.37% to 0.19%). Of the 50 seropositive antenatal women, 42 remained booked at our ART Centre for treatment. Thirty (71%) women are still adhered to taking ART. Of their 20 seropositive spouses, 14 (70%) have remained adhered to ART. Twenty-eight (93%) female patients on ART and 13 (93%) spouses on ART have suppressed viral loads. Two children of these seropositive mothers had tested HIV positive. ART adherence and suppressed viral load were seen in both seropositive children.

Conclusion

The study reflects a decline in antenatal seroprevalence in recent years in our region. The antenatal HIV prevalence trends have major implications on mother-to-child transmission and these positive antenatal cases serve as index cases bringing the testing opportunity for the so-called identified as the non-high-risk population. ART adherence of positive female patients, after the completion of the antenatal period, remains the challenge in our region, which requires improvement in the outreach activities and increased motivation and awareness of these patients regarding the importance of taking lifelong ART.

## Introduction

HIV is a major global public health issue. It has claimed 40.4 million lives up till now. In 2022, 0.63 million people lost their lives from HIV-related causes, and 1.3 million people were diagnosed with HIV. Owing to this global burden of HIV, the WHO, the Global Fund, and the Joint United Nations Programme on HIV/AIDS (UNAIDS) are working toward ending the HIV epidemic by 2030, which is the Sustainable Development Goal Target 3.3 [1,2]. The UNAIDS target is that by 2025, 95% of all people living with HIV (PLHIV) should have a diagnosis, 95% of those should be taking lifesaving anti-retroviral treatment (ART), and 95% of PLHIV on treatment should achieve a suppressed viral load for the benefit of the person’s health and for reducing onward HIV transmission. In 2022, the progress toward these 95-95-95 targets was 86%, 89%, and 93%, respectively as per the global data. Also, the 95-95-95 strategy is that 95% of reproductive-age women have their needs of HIV and sexual and reproductive health services met, with 95% of pregnant and breastfeeding women living with HIV having suppressed viral loads and 95% of HIV-exposed children tested by 2025 [1,3]. Global health sector strategies (GHSS) on HIV, viral hepatitis, and sexually transmitted infections for the period 2022-2030 guide to generate, analyze, and use evidence and data, with disaggregation by sex, age, and other relevant population characteristics. Taking into consideration the epidemiological, technological, and contextual shifts of previous years, this knowledge should be fostered across the disease areas, in a disease-country-specific manner and to leverage innovations and actions for ending the HIV epidemic [4]. The HIV statistics show that the Southeast Asian region has a decreasing trend in the number of PLHIV, HIV infection rate, HIV-related deaths at all ages, and an increasing trend of PLHIV receiving antiretroviral therapy from 2010 to 2022 [5]. The HIV epidemic in Gujarat state is pooled from other high-prevalence states of India, that are connected by highways, halt points of truck drivers with centers of commercial sexual services, and industrial belts that foster the migration of laborers from different states. Our hospital caters to a large number of antenatal out-state migratory populations, as Morbi district is the industrial hub of Gujarat state in India [6]. In this context, in our study, we intend to know the prevalence rate of HIV in antenatal women attending our hospital in the antenatal clinic and labor room and to know its trend over the last nine years. This trend over the years would help us understand the epidemiological pattern of HIV in our region to take targeted actions.

Because HIV infection is incurable, effective HIV prevention, diagnosis, treatment, and care, including for opportunistic infections, enables people living with HIV to lead long and healthy lives. Timely diagnosis and treatment are crucial to decrease HIV transmission in the community and decrease the morbidity of these patients. To increase the outreach of the diagnosis, the high-risk and vulnerable populations require enhanced testing. The effective “triple elimination initiative” recommended by WHO includes: testing for HIV in antenatal care clinics, prompt interventions to treat positive tested women to prevent transmission of the infection to their children, counseling for women and their partners to adopt practices to reduce transmission risk and ensure necessary treatment, safe institutional delivery, proper follow-up of exposed infants, optimal infant feeding practices, and lifelong treatment for mothers living with HIV [7]. The final mother-to-child transmission rate including the breastfeeding period in India was 19.9% in the year 2022 against the intended target of 5% or less [8]. The second aim of the study was to assess the implications of HIV testing in the prevention of transmission and management of the infection. Hence, the ART adherence and viral load suppression targets presently, of these seropositive women and their seropositive spouses and children, were analyzed.

## Materials and methods

The study was a retrospective study carried out in a tertiary care hospital in the western region of India. The study was approved by the Institutional Ethics Committee of GMERS (Gujarat Medical Education & Research Society) Medical College, Morbi, affiliated with the Civil Hospital, Morbi. The data were collected from the Prevention of Parent to Child Transmission (PPTCT) Centre and Anti-Retroviral Treatment (ART) Centre of the hospital for a period of nine years from January 2015 to December 2023. All the antenatal women attending the antenatal clinic and those admitted to the labor room are counseled for HIV testing under the PPTCT program in our hospital. Any antenatal woman who doesn’t wish to get tested can opt-out. The samples are tested according to the National AIDS Control Organisation (NACO) guidelines of India. Those antenatal women who turn out positive, their spouses, and children are tested for HIV. The data of the total number of antenatal women counseled (N) and tested for HIV during this period were collected. Data were collected for HIV-positive results (Np) and the prevalence of HIV in antenatal women was determined over these nine years. The trend of the prevalence over these years was analyzed.

The confidentiality and anonymity of the patients’ HIV data were maintained during the data collection process. Further data was retrieved from the ‘White Card’ - Patient Treatment Record from the ART Centre. The study population for this part of our study was the HIV-positive women who tested positive antenatally during the study period of nine years, and their seropositive family members - spouse and children. From these seropositive women (Np) and their seropositive spouses (Nps) and children (Npc), those transferred out to other ART Centres after the enrolment in our ART Centre were excluded from the study. The treatment data were collected to determine the ART adherence and suppressed viral load status. ART adherence refers to those patients who are on ART and are not lost to follow-up. Of those who are on ART, the data on their last viral load was studied. Suppressed viral load refers to viral load less than or equal to 1000 copies/mL.

The data was compiled and analyzed in a Microsoft Excel Sheet (Microsoft Corporation, Redmond, WA, USA). Frequency and percentage proportions were used for data representation and result interpretation.

## Results

A total of 22,584 (N), antenatal patients attending the antenatal clinic and labor room at Civil Hospital Morbi were counseled for HIV testing over a period of nine years and were tested for HIV. Of these, 50 (Np) antenatal patients were positive, giving an overall seroprevalence of 0.22 % over the nine-year study period. Table 1 shows the annual prevalence of HIV over nine years.

**Table 1:** Seroprevalence of HIV in antenatal women over nine years

Year	Total Antenatal Women Counselled for HIV Testing	Total Antenatal Women Tested for HIV	HIV Positive Antenatal Patients	HIV Prevalence in Antenatal Women
2015	1709	1709	4	0.23
2016	2182	2182	2	0.09
2017	1938	1938	5	0.25
2018	2086	2086	4	0.19
2019	2437	2437	7	0.28
2020	1874	1874	7	0.37
2021	2191	2191	6	0.27
2022	2884	2884	7	0.24
2023	4051	4051	8	0.19
Total	22584 (N)	22584	50 (Np)	0.22

Table 2 shows the frequency of seropositive antenatal patients, their spouses, and children who were tested for HIV subsequently, the number of those who were lost to follow-up for ART, and the proportion of these patients on ART at the time of data retrieval and with a suppressed viral load of less than 1000 copies/mL. With 20 (Nps) spouses testing seropositive, the spouse seroprevalence rate (Nps/Np) was 40% in our study group. These 50 seropositive mothers had 73 children; 24 children from previous pregnancies and 49 children from the index pregnancy at the time of HIV diagnosis. All 73 children of these PLHIV mothers had been tested for HIV in the hospital in the last nine years of the study period. Of the 50 children born from the pregnancies during which HIV diagnosis was done, one was a stillbirth. Of the rest, 49 live births, all had tested negative, as ART was started in these positive mothers antenatally, and the necessary prophylactic measures were taken for the prevention of mother-to-child transmission. All 50 antenatal patients were on ART during the entire antenatal period, however, 12 were lost to follow-up at various intervals after delivery. Of the 24 children born to these mothers from previous pregnancies before the diagnosis of HIV-positive status, two (Npc) turned out to be positive.

**Table 2:** Trend toward the anti-retroviral treatment adherence and viral load component status of the “95-95-95” strategy in our study subjects ART: anti-retroviral treatment; Suppressed viral load: viral load of less than or equal to 1000 copies/mL

	Tested HIV positive	Number of positive HIV patients transferred out to other ART centers (Excluded)	Number of positive HIV patients lost to follow-up	Number of the HIV-positive patients on ART (%)	Number of the HIV positive patients on ART and having suppressed viral load (%)
Antenatal women	50 (Np)	8	12	30 (71%)	28 (93%)
Spouses of these HIV-positive women	20 (Nps)	0	6	14 (70%)	13 (92.8%)
Children of these HIV-positive mothers	2 (Npc)	0	0	2 (100%)	2 (100%)

## Discussion

**Figure 1:**
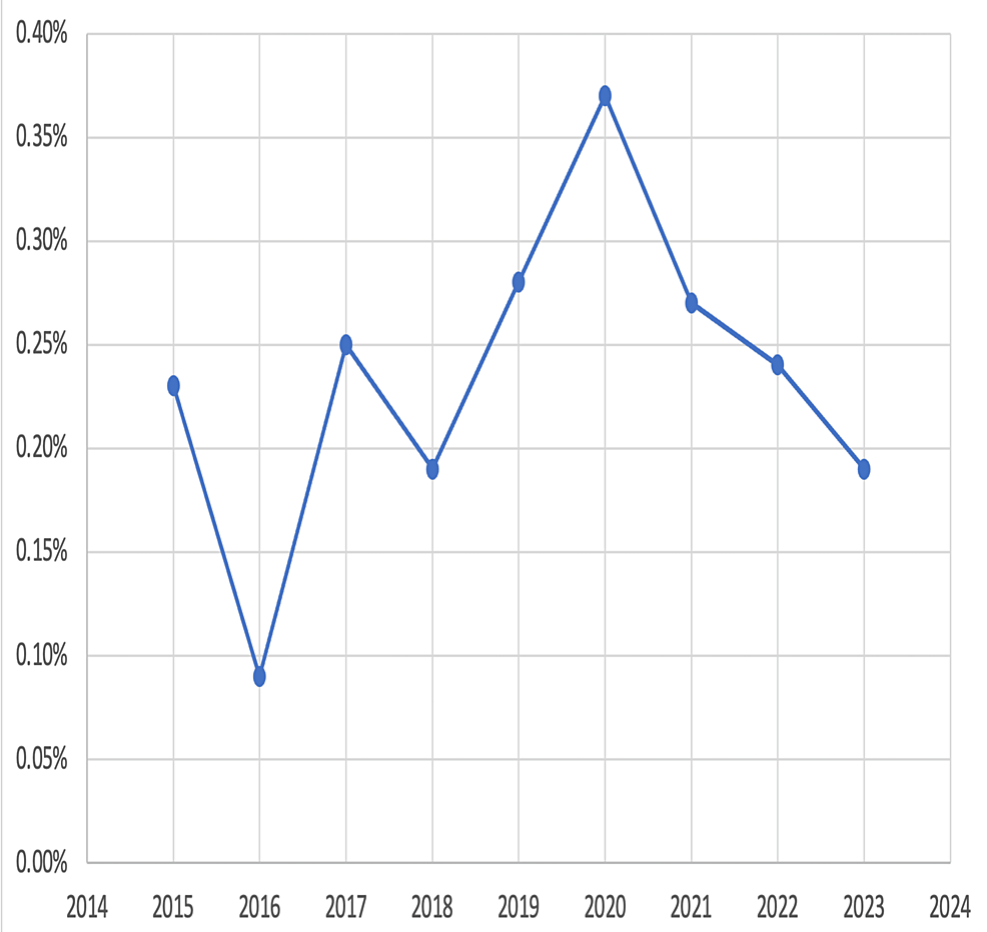
Trend of annual HIV prevalence in antenatal women from the year 2015 to 2023

Consistent and extensive data collection is required to determine the trend of HIV prevalence so as to have focused and effective planning and execution of HIV epidemic elimination policy aligned to the Global HIV strategies. Figure 1 shows the trend of HIV prevalence in our study in antenatal women.

The mean prevalence of HIV in our study over nine years was 0.22 %, as compared to the regional Gujarat state's prevalence of HIV in antenatal patients, which was 0.27 %, and a national seroprevalence of 0.22% [9]. The seroprevalence of 0.22% in our study is lower compared to similar studies done in Pune and Andhra Pradesh where the overall prevalence was higher being 0.6% and 0.30%, respectively [10,11]. Since 2002 till 2021, there has been a declining trend of HIV prevalence in the antenatal women in India and Gujarat state. Though from 2020 till 2023, there has been a declining trend of HIV in antenatal patients in our study, there has not been any consistent unidirectional trend over nine years of the study period [9]. There was an increase in the HIV prevalence in the 2020 COVID period, being 0.37% in our study. A similar higher trend was found in a study done in South Africa [12]. In our study, it was found that the adherence to ART in antenatal positive patients was 71% and in their seropositive spouses was 70%, which is less than the national and Gujarat state data of 84% [13]. This highlights the need for focused activities to raise awareness of these patients to improve their adherence to ART and increase outreach activities to trace these defaulting patients in the community. Poor adherence means fewer patients on ART with high viral loads, who remain the source of HIV transmission in the community. Poor adherence also reflects a casual attitude toward the disease and less resort to transmission-reducing behaviors by such patients. Also, it was noted that all 50 seropositive women remained adherent to ART during the antenatal period, but 12 became non-adherent postnatally (lost to follow-up). Hence, ART adherence postnatally and in later life is a challenge as compared to the antenatal period, which was also found in similar studies from Russia and various other countries [14,15]. All the children born of the index pregnancy, when the testing was done, tested negative for HIV, as ART was started during the antenatal period and necessary measures for the prevention of mother-to-child transmission were taken. Ninety-three percent of these female patients and their spouses on ART are now having suppressed viral load against the "95" target. Proper compliance and regularity of taking the medications, addressing the side effects, and addressing the drug resistance can help more patients on ART to have suppressed viral loads. The 2021 National data of India and Gujarat state data suggest that 85% and 84% of patients on ART have suppressed viral loads, respectively [13]. Against the 95% target of HIV-exposed children to be tested, by UNAIDS [3], 100% of the HIV-exposed children in our study were tested for HIV. Our study ascertains the conclusion of the study of Zambia, as there could be geospatial patterns toward the progress to the “95-95-95” targets, and there is a need to understand these local patterns to have regional policies to achieve zero transmission for the ending of the HIV epidemic [16].

The limitation of our study was that since the study was retrospective in nature, we could assess only the ART and viral suppression components of 95-95-95 targets for the HIV elimination strategy. It was not feasible to assess the first component, namely, the proportion of people living with HIV having awareness of HIV-positive diagnoses.

## Conclusions

The prevalence of HIV in antenatal women from our local region is similar to that of our country. However, the antenatal prevalence trend needs to be assessed regularly in the future to ensure timely actions are taken toward ending the HIV epidemic. Identifying HIV-positive antenatal patients provides the opportunity to trace their spouses and their children for the diagnosis and treatment of HIV as well as to prevent mother-to-child transmission. The ART adherence of positive female patients, after the completion of the antenatal period, remains a challenge in our region, requiring improvement in outreach activities and an increased awareness and motivation in these patients regarding the importance of taking lifelong ART.
